# Halfway rotational atherectomy for calcified lesions: Comparison with conventional rotational atherectomy in a propensity-score matched analysis

**DOI:** 10.1371/journal.pone.0219289

**Published:** 2019-07-05

**Authors:** Kenichi Sakakura, Yousuke Taniguchi, Kei Yamamoto, Hiroshi Wada, Shin-ichi Momomura, Hideo Fujita

**Affiliations:** Division of Cardiovascular Medicine, Saitama Medical Center, Jichi Medical University, Saitama City, Japan; Osaka University Graduate School of Medicine, JAPAN

## Abstract

**Background:**

The incidence of severe complications such as burr entrapment or perforation is considerable with rotational atherectomy (RA). Halfway RA is a novel strategy, in which an operator does not advance the burr to the end of a continuous calcified lesion, and performs balloon dilatation to treat the remaining part of the calcified lesion. The purpose of this study was to compare complications after halfway and conventional RA.

**Methods:**

We included 307 consecutive lesions that were divided into a conventional RA group (n = 244) and halfway RA group (n = 63). In analysis 1, the incidence of complications was compared between the conventional RA and halfway RA groups. Propensity-score matching was used to match the intentional halfway RA and conventional RA. In analysis 2, the incidence of complications was compared between the matched conventional RA and intentional halfway RA groups.

**Results:**

Burr entrapment (0.4%) and major perforation (0.8%) were observed in the conventional RA group, whereas there was no burr entrapment or perforation in the halfway RA group. The success rate of halfway RA was 90.5%, which required switching from halfway RA to conventional RA. The incidences of slow flow and periprocedural myocardial infarction with slow flow were similar between the intentional halfway RA and matched conventional RA groups.

**Conclusions:**

There was no burr entrapment or vessel perforation following halfway RA. The incidences of slow flow and periprocedural myocardial infarction were similar between the intentional halfway RA and the matched conventional RA, indicating the safety of halfway RA.

## Introduction

Although rotational atherectomy (RA) is necessary for severely calcified coronary lesions [1], the incidence of complications is greater in percutaneous coronary intervention (PCI) with RA than in PCI without RA [2–4]. Of note, the incidence of cardiac tamponade was more than 5 times greater in PCI with RA than in PCI without RA [2], suggesting a greater risk of vessel perforation in RA. Moreover, specific complications such as slow flow and burr entrapment is sometimes observed during RA [5–8]. Early studies including large registries reported the risk factors for complications in RA such as emergent PCI, hemodialysis, and previous myocardial infarction [2, 3, 9]. However, techniques or strategies to prevent such complications have not been fully described in the literature, partly because it may be difficult to collect data on specific techniques or strategies in large registries. In fact, we previously investigated the possibility that the low-speed RA decrease the incidence of slow flow, but the incidence of slow flow was similar between the low-speed and high-speed RA [8].

We previously reported an original strategy which we called “halfway RA”, for a severely calcified angulated lesion in a case report [10]. In halfway RA, the operator does not advance the RA burr beyond the angle within the lesion to avoid burr entrapment or vessel perforation, and balloon dilatation is performed beyond the angle after RA [10]. We hypothesized that halfway RA is safe and feasible for high-risk calcified lesions such as an angulated lesion or diffuse long lesion. The purpose of this study was to compare the incidence of complications after halfway RA and conventional RA for calcified coronary lesions.

## Methods

### Study design

**Fig 1:**
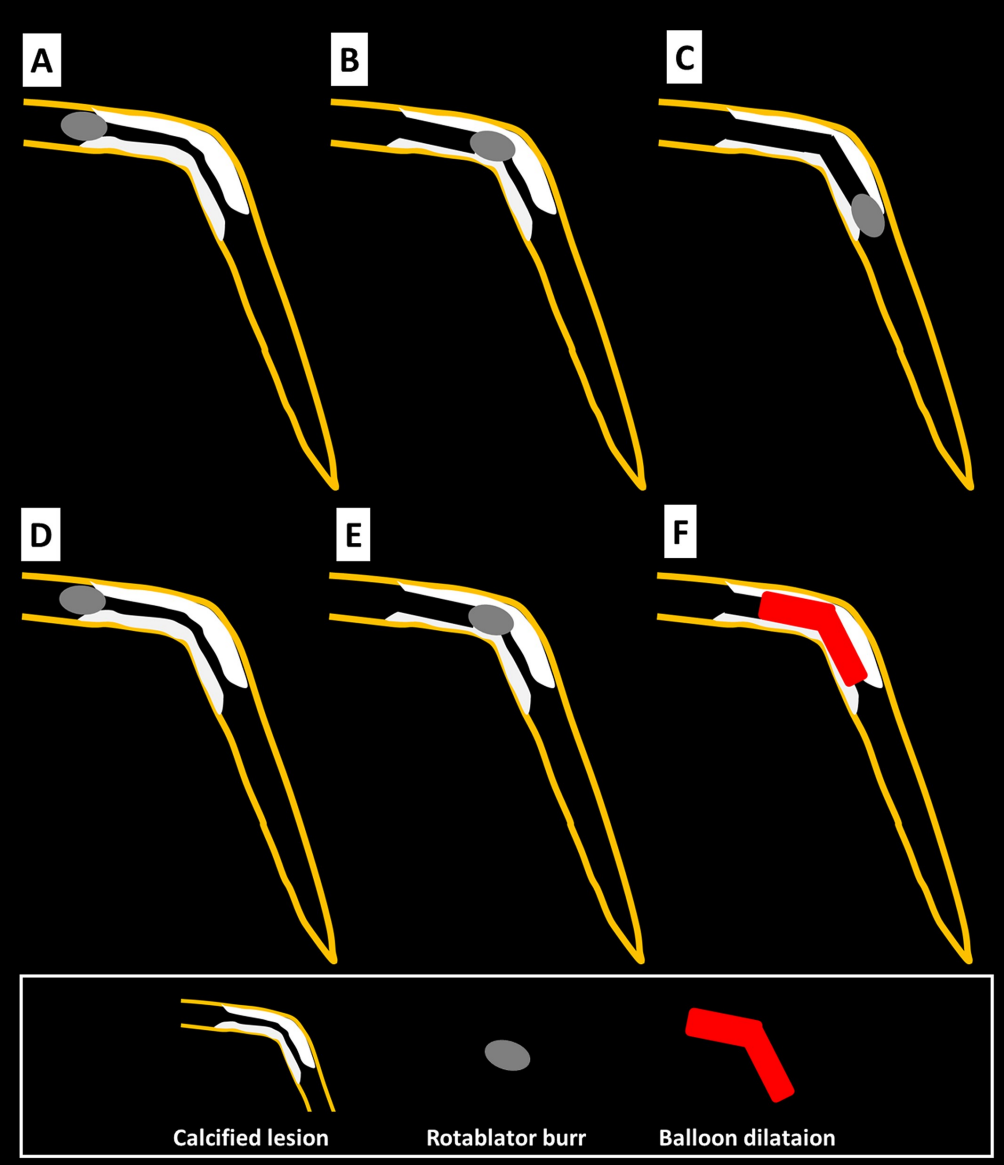
The scheme of conventional rotational atherectomy and halfway rotational atherectomy. Panels (A), (B), and (C) illustrate the conventional rotational atherectomy, whereas panels (D), (E), and (F) illustrate the halfway rotational atherectomy. (A): The burr positioned just before the calcified lesion. (B): The burr ablated the proximal segment of the calcified lesion. (C): The burr ablated the full segment of the calcified lesion. (D): The burr positioned just before the calcified lesion. (E): The burr ablated the proximal segment of the calcified lesion. (F): Balloon dilatation was performed for the rest of the calcified lesion.

This was a retrospective, single-center study. We included 307 consecutive coronary lesions that were treated by RA in our institution during the period from November 2014 to May 2018. Indications for RA in our institution are: 1) moderately or severely calcified lesions based on angiography, 2) diffuse lesions expected to be difficult to stent, and 3) ostial lesions [5, 8]. Halfway RA was defined as a procedure in which the operator did not advance the RA burr to the end of a continuous calcified lesion, whereas conventional RA was defined as a procedure in which the operator advanced the RA burr to the end of a continuous calcified lesion (Fig 1). We previously performed a prospective, randomized study comparing high-speed RA and low-speed RA in our catheter laboratory from November 2014 to February 2016 [8], and we recorded every burr movement during RA by fluoroscopic image storage since November 2014. This allowed us to retrospectively judge whether each procedure was halfway RA or conventional RA.

**Fig 2:**
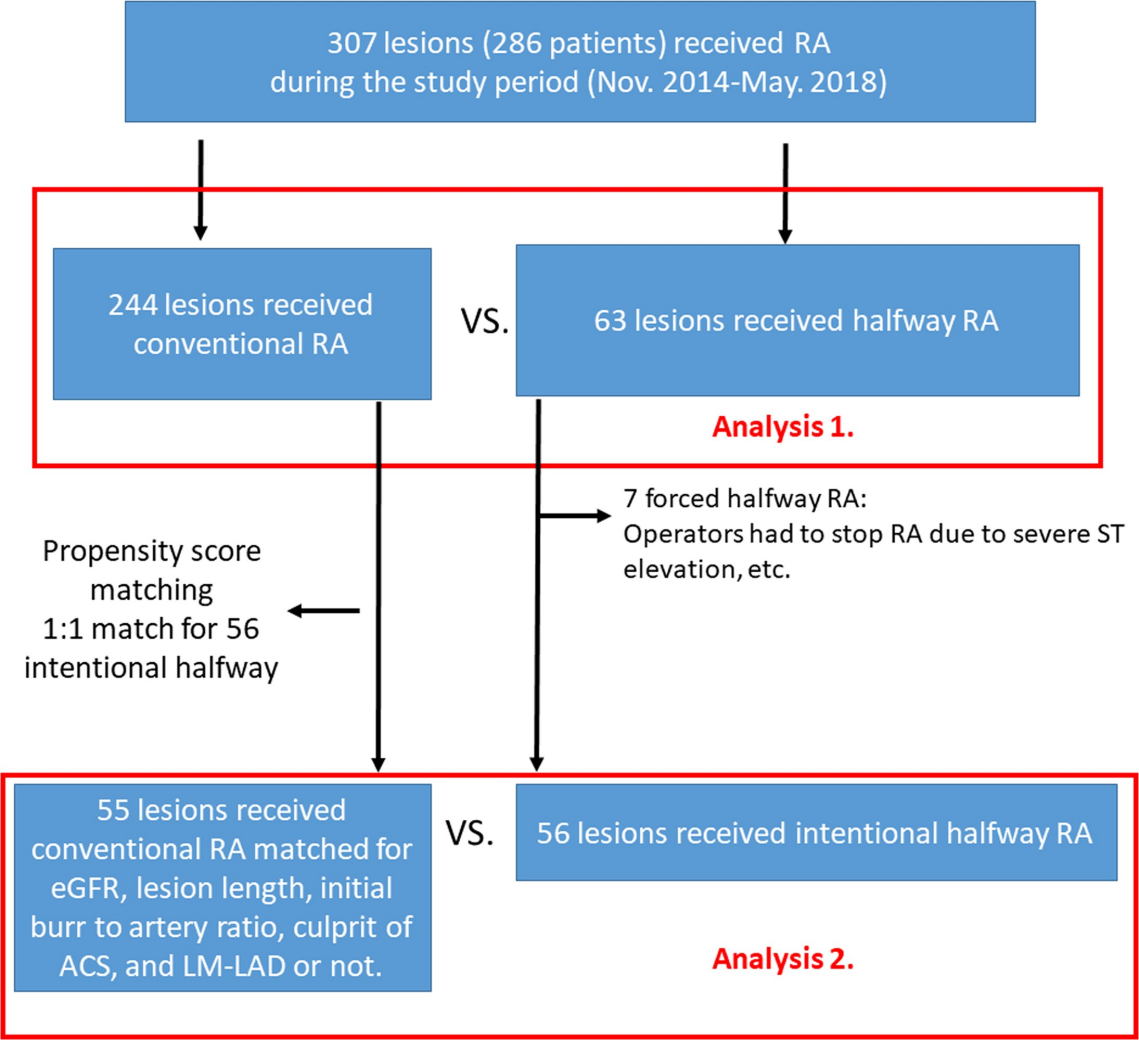
Study flow chart. Abbreviations: RA = rotational atherectomy, eGFR = estimated glomerular filtration rate, ACS = acute coronary syndrome, LM-LAD = left main-left anterior descending artery.

In analysis 1, the study lesions were divided into the conventional RA group (n = 244) and the halfway RA group (n = 63). Clinical characteristics and the incidence of complications were compared between the conventional RA and halfway RA groups. Halfway RA was performed intentionally in most cases (intentional halfway RA); however, operators occasionally were forced to perform halfway RA because of severe ST-elevation during RA (forced halfway RA). As mild ST-elevation is common during RA, mild ST-elevation would not force to halfway RA. In analysis 2, we excluded forced halfway RA cases (n = 7) to determine the utility of intentional halfway RA (n = 56). Propensity-score matching was performed to match the intentional halfway RA group and the conventional RA group for estimated glomerular filtration rate (eGFR), lesion length, initial burr-to-artery ratio, culprit lesion in acute coronary syndrome, and left main (LM)-left anterior descending artery (LAD) disease. The details of propensity-score matching are described in the section on statistical analysis. One-to-one propensity-score matching resulted in 56 lesions in the intentional halfway RA group and 55 lesions in the conventional RA group. In analysis 2, clinical characteristics and the incidence of complications were compared between the matched conventional RA and intentional halfway RA groups. The study flow chart is shown in Fig 2. The study was approved by the institutional review board of Saitama Medical Center, Jichi Medical University, and written informed consent was waived because of the retrospective study design.

### Rotational atherectomy

RA was performed using standard techniques except for halfway RA[5], and RA in our catheter laboratory is described in elsewhere [5, 8, 11]. The lesion was crossed with a 0.014 inch conventional guidewire, which was exchanged with a 0.009 inch RotaWire floppy or RotaWire extra support guidewire (Boston Scientific, Natick, MA, USA) using a microcatheter. The RA burr was subsequently advanced over the wire to a position proximal to the lesion. The rotational speed was set at the conventional range (140,000–190,000 rpm) with the burr proximal to the lesion, and 100 lesions were randomly allocated to 140,000 rpm or 190,000 rpm [8]. The burr was activated and moved forward with a slow pecking motion. Each run time was < 30 seconds, and care was taken to avoid a decrease in rotational speed > 5000 rpm. The initial burr size was either 1.25 mm or 1.5 mm, which is supported by the European expert consensus on RA [12]. After the burr passed the lesion, the burr was pulled out using the dynaglide mode or trapping balloon technique [13]. The presence of coronary flow was confirmed by injecting sufficient contrast medium immediately after the burr was pulled out. Following RA, balloon dilatation was performed using a non-compliant balloon to facilitate stent implantation.

### Endpoints

The endpoint of this study was the occurrence of complications related to RA. We collected information on the following complications: slow flow just after RA, Thrombolysis in Myocardial Infarction (TIMI) flow grade just after RA, burr entrapment, vessel perforation (any), vessel perforation (type III) due to the burr, vessel perforation (type II) due to the guidewire, and periprocedural myocardial infarction with slow flow. Slow flow just after RA was defined as slow or absent distal runoff (TIMI flow grade ≤ 2) [11, 14]. Periprocedural myocardial infarction was defined as an increase in creatine kinase (at least a threefold increase above the normal upper limit) [5, 8].

### Definitions

Clinical criteria were defined as follows. eGFR was calculated using the MDRD formula [15]. ACS was defined as ST-segment elevation myocardial infarction, non-ST-segment elevation myocardial infarction, or unstable angina. The reference diameter, lesion length, mean stent diameter, and minimum stent diameter were calculated by quantitative coronary angiographic analysis. Offline, computer-based software QAngio XA 7.3 (MEDIS Imaging Systems, Leiden, The Netherlands) was used for quantitative coronary angiographic analysis. The burr-to-artery ratio was defined as the burr size divided by the reference diameter.

### Statistical analysis

Data are presented as a percentage for categorical variables and the mean ± SD for continuous variables. The Wilk-Shapiro test was performed to determine if the continuous variables were normally distributed. Normally distributed continuous variables were compared between groups using an unpaired Student’s t test. Otherwise, continuous variables were compared using a Mann-Whitney U test. Categorical data were compared using a χ^2^ test. Propensity score matching was applied to compare the incidence of complications between the intentional halfway RA group and the matched conventional RA group. First, a logistic regression analysis was performed to calculate the propensity score using the full database except for forced halfway RA cases. In this model, the intentional halfway RA was set as a dependent variable, whereas parameters that were clinically relevant for complications in RA, such as eGFR, lesion length, initial burr-to-artery ratio, culprit of acute coronary syndrome, and LM-LAD disease, were set as independent variables [2, 8]. Since the number of dependent variable (the intentional halfway RA) was 56, the maximum number of independent variables should be 5 [16, 17]. Therefore, we selected the above 5 variables as independent variables. For matching, the match tolerance was set as a width of 0.25 multiplied by the SD of the propensity score distribution [18, 19]. Case-control matching found 55 fuzzy matches with maximizing matching performance. Thus, 56 intentional halfway RA lesions and 55 matched conventional RA lesions were selected for analysis 2. All reported P-values were determined by two-sided analysis, and P-values < 0.05 were considered significant. All analyses including the propensity-score matching were performed with IBM SPSS statistics version 25 (Chicago, IL, USA).

## Results

During the study period (from November 18, 2014 to May 31, 2018), seven operators in our institution performed RA; however, most cases (n = 304, 99%) were supervised by a single experienced operator (K. Sakakura). As a total of 2635 PCI were performed during the study period, the percentage of RA was 11.7%. Patient, lesion, and procedural characteristics between the groups are summarized in Table 1. The lesion length was significantly longer in the halfway RA group (29.19±14.15 mm) than in the conventional RA group (23.95±15.50 mm) (P = 0.004). The lesion angle was greater in the halfway RA group than in the conventional RA group (P <0.001). The initial burr-to-artery ratio was significantly less in the conventional RA group (0.57±0.14) than in the halfway RA group (0.64±0.15) (P <0.001).

**Table 1:** Comparison of patient, lesion, and procedural characteristics between the conventional RA and halfway RA groups.

Variables	All (n = 307)	Conventional RA group (n = 244)	Halfway RA group (n = 63)	P value
Patient characteristics				
Age (years)	73.8±7.9	73.5±8.2	74.8±6.3	0.45
Men—n (%)	234 (76.2)	186 (76.2)	48 (76.2)	1.00
Overweight (body mass index ≥25 kg/m^2^)—n (%)	72 (23.5)	61 (25.0)	11 (17.5)	0.21
Hypertension—n (%)	291 (94.8)	230 (94.3)	61 (96.8)	0.41
Diabetes mellitus—n (%)	157 (51.3)	129 (53.1)	28 (44.4)	0.22
Hyperlipidemia—n (%)	287 (93.5)	224 (91.8)	63 (100)	0.02
Current smoker—n (%)	51 (16.6)	40 (16.4)	11 (17.5)	0.84
Chronic renal failure (creatinine >2mg/dl)—n (%)	70 (22.8)	56 (23.0)	14 (22.2)	0.90
Estimated GFR (mL/mn/1.73m^2^)	68.4±39.6	67.9±39.5	70.1±39.9	0.59
Chronic renal failure on hemodialysis—n (%)	60 (19.5)	49 (20.1)	11 (17.5)	0.64
Statin treatment—n (%)	280 (91.2)	218 (89.3)	62 (98.4)	0.02
Lesion characteristics				
Culprit lesion in acute coronary syndrome—- n (%)	55 (17.9)	45 (18.4)	10 (15.9)	0.64
Target coronary artery				0.69
Left main- left anterior descending artery—n (%)	226 (73.6)	177 (72.5)	49 (77.8)	
Left circumflex artery—n (%)	13 (4.2)	11 (4.5)	2 (3.2)	
Right coronary artery—n (%)	68 (22.1)	56 (23.0)	12 (19.0)	
Reference diameter (mm)	2.42±0.62	2.47±0.63	2.22±0.52	0.003
Lesion length (mm)	25.03±15.36	23.95±15.50	29.19±14.15	0.004
Mean stent diameter (mm)	2.96±0.41 (n = 303)	3.00±0.41	2.82±0.38	0.001
Minimum stent diameter (mm)	2.34±0.48 (n = 303)	2.40±0.49	2.11±0.36	<0.001
Lesion angle				<0.001
Mild angulation (<30°)	162 (52.8)	145 (59.4)	17 (27.0)	
Moderate angulation (30–60°)	112 (36.5)	81 (33.2)	31 (49.2)	
Severe angulation (≥60°)	33 (10.7)	18 (7.4)	15 (23.8)	
Procedural characteristics				
Guiding catheter size and system				0.50
6Fr—n (%)	4 (1.3)	4 (1.6)	0 (0)	
7Fr—n (%)	288 (93.8)	229 (93.9)	59 (93.7)	
8Fr—n (%)	15 (4.9)	11 (4.5)	4 (6.3)	
Intra-aortic balloon pump support—n (%)	36 (11.7)	32 (13.1)	4 (6.3)	0.14
Guidewire used during rotational atherectomy				<0.001
RotaWire floppy—n (%)	249 (81.1)	205 (84.0)	44 (69.8)	
RotaWire extra support—n (%)	35 (11.4)	29 (11.9)	6 (9.5)	
Guidewire switch (floppy ↔ extra support)—n (%)	23 (7.5)	10 (4.1)	13 (20.6)	
Number of burrs used	1.2±0.4	1.2±0.4	1.1±0.5	0.29
Initial burr size (mm)	1.34±0.12	1.33±0.12	1.35±0.12	0.21
Final burr size (mm)	1.40±0.21	1.39±0.21	1.40±0.19	0.49
Initial burr-to-artery ratio	0.59±0.15	0.57±0.14	0.64±0.15	<0.001
Final burr-to-artery ratio	0.61±0.16	0.60±0.16	0.66±0.17	0.003
Total run time (seconds)	102.8±69.7	99.1±68.8	117.3±72.0	0.03
Mean single run time (seconds)	15.5±4.4	15.4±4.4	15.6±4.3	0.75
Mean rotational speed (x 1000 rpm)	166.9±17.1	166.2±17.6	169.7±14.7	0.42
Maximum speed reduction during rotational atherectomy (rpm)	5900±4562 (300)	5954±4943 (n = 239)	5689±2598 (n = 61)	0.40
Systolic blood pressure just before rotational atherectomy	149.3±25.6	150.7±25.6	143.8±24.9	0.04
Diastolic blood pressure just before rotational atherectomy	75.3±13.5	75.8±13.4	73.5±13.7	0.20
Heart rate just before rotational atherectomy (per minute)	70.1±12.5	70.3±12.8	69.2±11.3	0.98
Final procedure				0.27
Rotational atherectomy + balloon—n (%)	3 (1.0)	3 (1.2)	0 (0)	
Rotational atherectomy + bare-metal stent—n (%)	5 (1.6)	4 (1.6)	1 (1.6)	
Rotational atherectomy + drug-eluting stent—n (%)	296 (96.4)	235 (96.3)	61 (96.8)	
Rotational atherectomy + covered stent for perforation—n (%)	2 (0.7)	2 (0.8)	0 (0)	
Unsuccessful procedure—n (%)	1 (0.3)	0 (0)	1 (1.6)	

Data are expressed as the mean±SD or number (percentage). A Student’s t test was used for normally distributed continuous variables, a Mann-Whitney U test was used for abnormally distributed continuous variables, and a chi-square test was used for categorical variables. Abbreviations: GFR = glomerular filtration rate

The comparison of complications between the groups is shown in Table 2. The incidence of slow flow was significantly greater in the halfway RA group (38.1%) than in the conventional RA group (15.6%) (P <0.001). Burr entrapment or vessel perforation (type III) due to the burr was rarely observed in this study. The details of a burr entrapment case and a vessel perforation case are published elsewhere [7, 20]. Of note, there was no burr entrapment or vessel perforation in the halfway RA group.

**Table 2:** Comparison of complications between the conventional RA and halfway RA groups.

Complications	All (n = 307)	Conventional RA group (n = 244)	Halfway RA group (n = 63)	P value
Slow flow just after RA—n (%)	62 (20.2)	38 (15.6)	24 (38.1)	<0.001
TIMI flow grade just after RA				0.001
TIMI 0 flow—n (%)	3 (1.0)	2 (0.8)	1 (1.6)	
TIMI 1 flow—n (%)	22 (7.2)	13 (5.3)	9 (14.3)	
TIMI 2 flow—n (%)	37 (12.1)	23 (9.4)	14 (22.2)	
TIMI 3 flow—n (%)	245 (79.8)	206 (84.4)	39 (61.9)	
Periprocedural myocardial infarction with slow flow—n (%)	9 (2.9)	5 (2.0)	4 (6.3)	0.07
Burr entrapment—n (%)	1 (0.3)	1 (0.4)	0	0.61
Vessel perforation (any)—n (%)	4 (1.3)	4 (1.6)	0	0.31
Vessel perforation (type III) due to Burr—n (%)	2 (0.7)	2 (0.8)	0	0.47
Vessel perforation (type II) due to guidewire—n (%)	2 (0.7)	2 (0.8)	0	0.47

Data are expressed as the number (percentage). A chi-square test was used to compare the two groups. Abbreviations: TIMI = Thrombolysis in Myocardial infarction.

Procedural outcomes and reasons for halfway RA are shown in Table 3. The success rate of halfway RA was 90.5%. Unsuccessful balloon dilatation following halfway RA was observed in 6 lesions (9.5%). Five of those six cases required switching from halfway RA to conventional RA, and final procedure success was achieved in those 5 lesions. In one of those six cases, we could not deliver any devices following halfway RA, which showed chronic total occlusion with severe calcification. Final procedure success was not achieved in that lesion. The most dominant reason for halfway RA was an angle within the lesion (61.9%), and operators had to stop RA in the middle of the calcified lesion (forced halfway RA) in 7 lesions (11.1%). In those 7 forced halfway RA, successful halfway RA was achieved in 6 of these 7 lesions (86%), and 1 lesion (14%) required switching from halfway RA to conventional RA.

**Table 3:** Procedural outcomes of halfway rotational atherectomy and reasons for halfway rotational atherectomy.

Procedural outcomes of halfway RA (n = 63)	
Successful balloon dilatation following halfway RA—n (%)	57 (90.5)
Unsuccessful balloon dilatation following halfway RA, and switched to conventional RA—n (%)	5 (7.9)
Unsuccessful delivery of any device following halfway RA—n (%)	1 (1.6)
Reason for halfway RA (n = 63)	
Diffuse long lesion—n (%)	12 (19.0)
Angle within the lesion—n (%)	39 (61.9)
Operator felt resistance within the lesion—n (%)	5 (7.9)
Forced halfway due to severe ST elevation, etc.—n (%)	7 (11.1)

Data are expressed as the number (percentage). Abbreviations: RA = rotational atherectomy.

Patient, lesion, and procedural characteristics between the propensity-score matched groups are summarized in Table 4. The eGFR, lesion length, initial burr-to-artery ratio, culprit lesion in acute coronary syndrome, and target coronary artery were not different between the matched groups, while the lesion angle was still greater in the intentional halfway RA group. A comparison of complications between the propensity-score matched groups is shown in Table 5. The incidences of slow flow, TIMI flow grades, and periprocedural myocardial infarction with slow flow were similar between the matched groups.

**Table 4:** Comparison of patient, lesion, and procedural characteristics between the matched conventional RA (n = 55) and intentional halfway RA groups (n = 56).

Variables	All (n = 111)	Matched conventional RA group (n = 55)	Intentional halfway RA group (n = 56)	P value
Patient characteristics				
Age (years)	74.1±6.7	73.4±7.0	74.8±6.3	0.36
Men—n (%)	87 (78.4)	44 (80.0)	43 (76.8)	0.68
Overweight (body mass index ≥25 kg/m^2^)—n (%)	19 (17.1)	11 (20.0)	8 (14.3)	0.42
Hypertension—n (%)	106 (95.5)	52 (94.5)	54 (96.4)	0.63
Diabetes mellitus—n (%)	54 (48.6)	30 (54.4)	24 (42.9)	0.22
Hyperlipidemia—n (%)	104 (93.7)	48 (87.3)	56 (100)	0.006
Current smoker—n (%)	18 (16.2)	9 (16.4)	9 (16.1)	0.97
Chronic renal failure (creatinine >2mg/dl)—n (%)	19 (17.1)	7 (12.7)	12 (21.4)	0.22
Estimated GFR (mL/mn/1.73m^2^)	71.4±35.5	71.1±30.4	71.7±40.2	0.72
Chronic renal failure on hemodialysis—n (%)	14 (12.6)	4 (7.3)	10 (17.9)	0.09
Statin treatment—n (%)	101 (91.0)	46 (83.6)	55 (98.2)	0.007
Lesion characteristics				
Culprit lesion in acute coronary syndrome—n (%)	13 (11.7)	5 (9.1)	8 (14.3)	0.40
Target coronary artery				0.34
Left main- left anterior descending artery—n (%)	88 (79.3)	45 (81.8)	43 (76.8)	
Left circumflex artery—n (%)	6 (5.4)	4 (7.3)	2 (3.6)	
Right coronary artery—n (%)	17 (15.3)	6 (10.9)	11 (19.6)	
Reference diameter (mm)	2.25±0.56	2.24±0.60	2.26±0.51	0.55
Lesion length (mm)	29.90±15.04	30.70±15.45	29.12±14.72	0.58
Mean stent diameter (mm)	2.88±0.42 (n = 110)	2.92±0.45	2.85±0.38 (n = 55)	0.54
Minimum stent diameter (mm)	2.25±0.56 (n = 110)	2.36±0.69	2.14±0.36 (n = 55)	0.047
Lesion angle				0.01
Mild angulation (<30°)	45 (40.5)	30 (54.5)	15 (26.8)	
Moderate angulation (30–60°)	47 (42.3)	19 (34.5)	28 (50.0)	
Severe angulation (≥60°)	19 (17.1)	6 (10.9)	13 (23.2)	
Procedural characteristics				
Guiding catheter size and system				0.05
6Fr—n (%)	2 (1.8)	2 (3.6)	0 (0)	
7Fr—n (%)	105 (94.6)	53 (96.4)	52 (92.9)	
8Fr—n (%)	4 (3.6)	0 (0)	4 (7.1)	
Intra-aortic balloon pump support—n (%)	11 (9.9)	7 (12.7)	4 (7.1)	0.33
Guidewire used during rotational atherectomy				0.02
RotaWire floppy—n (%)	88 (79.3)	49 (89.1)	39 (69.6)	
RotaWire extra support—n (%)	10 (9.0)	4 (7.3)	6 (10.7)	
Guidewire switch (floppy ↔ extra support)—n (%)	13 (11.7)	2 (3.6)	11 (19.6)	
Number of burrs used	1.1±0.4	1.1±0.3	1.1±0.5	0.51
Initial burr size (mm)	1.31±0.11	1.26±0.05	1.36±0.13	<0.001
Final burr size (mm)	1.35±0.18	1.30±0.14	1.41±0.19	<0.001
Initial burr-to-artery ratio	0.61±0.14	0.60±0.14	0.63±0.14	0.28
Final burr-to-artery ratio	0.63±0.16	0.61±0.16	0.65±0.17	0.20
Total run time (seconds)	115.4±55.3	123.0±45.5	108.0±62.9	0.02
Mean single run time (seconds)	17.6±4.2	20.1±2.6	15.1±3.9	<0.001
Mean rotational speed (x 1000 rpm)	167.0±19.9	163.7±23.6	170.2±14.9	0.91
Maximum speed reduction during rotational atherectomy (rpm)	5952±3534 (n = 105)	6333±4376 (n = 51)	5593±2484 (n = 54)	0.59
Systolic blood pressure just before rotational atherectomy	143.7±23.0	145.4±22.0	142.2±24.1	0.21
Diastolic blood pressure just before rotational atherectomy	74.1±12.7	74.7±12.0	73.5±13.5	0.29
Heart rate just before rotational atherectomy (per minute)	69.6±11.4	69.7±11.6	69.5±11.3	0.79
Final procedure				0.39
Rotational atherectomy + balloon—n (%)	0 (0)	0 (0)	0 (0)	
Rotational atherectomy + bare-metal stent—n (%)	2 (1.8)	1 (1.8)	1 (1.8)	
Rotational atherectomy + drug-eluting stent—n (%)	106 (95.5)	52 (94.5)	54 (96.4)	
Rotational atherectomy + covered stent for perforation—n (%)	2 (1.8)	2 (3.6)	0 (0)	
Unsuccessful procedure—n (%)	1 (0.9)	0 (0)	1 (1.8)	

Data are expressed as the mean±SD or number (percentage). A Student’s t test was used for normally distributed continuous variables, a Mann-Whitney U test was used for abnormally distributed continuous variables, and a chi-square test was used for categorical variables. Abbreviations: GFR = glomerular filtration rate.

**Table 5:** Comparison of complications between the matched conventional RA (n = 55) and intentional halfway RA (n = 56) groups.

Complications	All (n = 111)	Matched conventional RA group (n = 55)	Intentional halfway RA group (n = 56)	P value
Slow flow just after RA	35 (31.5)	18 (32.7)	17 (30.4)	0.79
TIMI flow grade just after RA				0.85
TIMI 0 flow	2 (1.8)	1 (1.8)	1 (1.8)	
TIMI 1 flow	12 (10.8)	5 (9.1)	7 (12.5)	
TIMI 2 flow	21 (18.9)	12 (21.8)	9 (16.1)	
TIMI 3 flow	76 (68.5)	37 (67.3)	39 (69.6)	
Periprocedural myocardial infarction with slow flow	6 (5.4)	3 (5.5)	3 (5.4)	0.98
Burr entrapment	0 (0)	0 (0)	0 (0)	-
Vessel perforation (any)	3 (2.7)	3 (5.5)	0 (0)	0.08
Vessel perforation (Type III) due to Burr	2 (1.8)	2 (3.6)	0 (0)	0.15
Vessel perforation (Type II) due to guidewire	1 (0.9)	1 (1.8)	0 (0)	0.31

Data are expressed as the number (percentage). A chi-square test was used to compare the two groups. Abbreviations: TIMI = Thrombolysis in Myocardial infarction.

## Discussion

Of 307 lesions treated by RA, halfway RA was performed in 63 lesions (20.5%). The success rate of halfway RA was 90.5% (57 lesions), and the switch from halfway RA to conventional RA was feasible after unsuccessful balloon dilatation in 5 of 6 lesions. Since lesion length, which is a strong determinant of slow flow during RA [8, 11], was significantly longer with halfway RA than with conventional RA, halfway RA seemed to be attempted when there were more complex lesions. Therefore, the incidence of slow flow was greater in the halfway RA group than in the conventional RA group. Nevertheless, burr entrapment or vessel perforation was not observed in the halfway RA group, suggesting the safety of halfway RA. Moreover, after propensity-score matching, the incidence of slow flow or periprocedural myocardial infarction with slow flow was similar between the intentional halfway RA and conventional RA groups. This suggested the utility of intentional halfway RA as an alternative method of conventional RA for high-risk calcified lesions.

Although many clinical trials have been published on RA, most of these trials compared RA with balloon angioplasty for the treatment of de novo coronary artery disease [21–24], in-stent restenosis [25, 26], or lesion preparation for drug-eluting stent implantation [27]. There were only a few studies on techniques or strategies for RA. Two studies compared a small-burr strategy (burr-to-artery ratio ≤ 0.7) with a large-burr strategy (burr-to-artery ratio > 0.7) [28, 29], and these studies reported the superiority of the small-burr strategy with fewer complications. We previously conducted a randomized study to compare the incidence of slow flow between low-speed (140,000 rpm) and high-speed (190,000 rpm) RA, and showed a similar incidence of slow flow between the two speeds [8]. To the best of our knowledge, there has been no study that investigated the association between complications and the method used to advance the RA burr.

It is important to address the reason that burr entrapment or vessel perforation was not observed with halfway RA in the present study. In general, the risk of burr entrapment or vessel perforation is greater in an angulated calcified lesion compared with a straight calcified lesion; therefore, the official product document of the Rotablator (Boston Scientific, Natick, MA, USA) warns of a greater risk of complications in an angulated lesion [30]. Several reports of burr entrapment or perforation following RA of angulated lesions have been published [6, 7, 31]. Since the shape of an RA burr is nearly ellipsoidal, the RA burr may not effectively advance beyond the angle [32], and operators may feel resistance in the middle of an angulated lesion. Therefore, there would be a greater risk of burr entrapment or perforation in the middle of an angulated lesion. On the other hand, since operators do not advance the burr beyond the middle of the angulated lesion in halfway RA, there would be a low risk of burr entrapment or perforation [32].

Although there was no guarantee that the calcified lesion could be dilated with a balloon following halfway RA, the success rate of halfway RA was high (90.5%) in the present study. Our results suggest that the extent of calcification (severity or eccentricity) is not equal within a calcified lesion. If halfway RA ablates the most severely calcified part of the lesion, other calcified parts of the lesion may dilate following balloon dilatation. Although intravascular ultrasound or optical coherence tomography before RA may show the distribution of calcification more precisely [33], the imaging catheter itself may not cross a severely calcified lesion before RA. Furthermore, the advantage of halfway RA was that switching from halfway RA to conventional RA is easy, because there would be less severe complications such as perforation or burr entrapment following halfway RA. Therefore, it would be a practical approach to try halfway RA first for severely calcified lesions.

Clinical implications for halfway RA should be further discussed. While a goal of RA is to modify the calcification to facilitate stent dilatation, another goal of RA is to finish procedures without severe complications such as vessel perforation or burr entrapment. RA operators should have several options when they feel difficulty to advance the RA burr in the calcified lesion, because most of severe complications occur after operators push the burr in spite of strong resistance. Downsizing the burr size is a standard option when operators feel strong resistance[12], and the exchange the RotaWire from floppy to extra support or from extra support to floppy may work because of different guidewire bias [33]. However, those options may not work for diffuse long lesions or acute angle lesions with severe calcification. If operators have enough experience in RA, they probably stop RA and switch to balloon dilatation to avoid severe complications (defensive RA or partial RA). The concept of halfway RA is similar to those defensive RA or partial RA, which were not systematically described in literatures. Therefore, halfway RA would bridge the technical gap between experienced senior RA operators and less experienced RA operators, which may decrease the overall incidence of severe complications during RA.

### Study limitations

Because our study was designed as a single-center, retrospective, observational study, there was a risk of patient selection bias and group-selection bias. The study population might not have been large enough to find a between-group difference in severe complications, such as burr entrapment or perforation, because these complications were rarely observed. Therefore, the fact that there were no severe complications in halfway RA does not mean that halfway RA could prevent severe complications. The overall incidence of slow flow (20.2%) in the present study was relatively higher than that in earlier studies [23, 34], which were conducted before the drug-eluting stent era. Lesion length, which is a strong determinant of slow flow after RA [8, 11], was much longer in the present study (25 mm) than in those studies (around 13mm) [23, 34]. Since we experienced a typical case of halfway RA in the middle of the study period [10], we selected halfway RA more frequently after that case, which may have caused a selection bias. Although one of the reasons for halfway RA was that the “operator felt resistance within the lesion (n = 5)”, halfway RA was not the only option when operators felt resistance within the lesion. Operators could also have exchanged the RotaWire from floppy to extra support or changed the burr size [32]. Although we proposed that the halfway RA is a reasonable option for angulated calcified lesions, there is not enough data to prove it in the present study. Furthermore, although we proposed halfway RA to prevent burr entrapment, there would be other options or techniques (i.e., pecking motion, short duration of ablation, short segment ablation during the runs) to avoid burr entrapment [12]. Finally, the minimum stent diameter was smaller in the halfway RA than in the conventional RA. Although the lesion complexity such as angulation might affect the less minimum stent diameter, insufficient ablation caused by halfway RA might cause the less minimum stent diameter. Future study is warranted to assess long-term outcomes in halfway RA.

## Conclusions

There was no burr entrapment or vessel perforation following halfway RA. The incidences of slow flow were similar between the intentional halfway RA and the propensity-score matched conventional RA groups, suggesting the utility of intentional halfway RA as an alternative method of conventional RA for high-risk calcified lesions.

## Supporting information

S1 File Analysis data (n = 307). (XLSX)

S2 File Analysis data (n = 111, after PS matching). (XLSX)
